# The Co-occurrence of NDM-5, MCR-1, and FosA3-Encoding Plasmids Contributed to the Generation of Extensively Drug-Resistant *Klebsiella pneumoniae*

**DOI:** 10.3389/fmicb.2021.811263

**Published:** 2022-01-03

**Authors:** Ying Zhou, Wenxiu Ai, Yanhua Cao, Yinjuan Guo, Xiaocui Wu, Bingjie Wang, Lulin Rao, Yanlei Xu, Huilin Zhao, Xinyi Wang, Fangyou Yu

**Affiliations:** ^1^Department of Clinical Laboratory Medicine, Shanghai Pulmonary Hospital, School of Medicine, Tongji University, Shanghai, China; ^2^Department of Respiratory Medicine, The First Affiliated Hospital of Wenzhou Medical University, Wenzhou, China; ^3^Department of Respiratory Intensive Care Unit, Shanghai Pulmonary Hospital, School of Medicine, Tongji University, Shanghai, China; ^4^Department of Laboratory Medicine, The First Affiliated Hospital of Wenzhou Medical University, Wenzhou, China

**Keywords:** *K. pneumoniae*, plasmid, *fosA3*, *bla*_NDM–5_, *mcr-1*, mobile elements

## Abstract

The rise and global dissemination of extensively drug-resistant (XDR) bacteria are often related to plasmid-borne mobile antimicrobial resistance genes. Notably, isolates having multiple plasmids are often highly resistant to almost all the antibiotics available. In this study, we characterized an extensively drug-resistant *Klebsiella pneumoniae* 1678, which exhibited high-level resistance to almost all the available antibiotics. Through whole-genome sequencing (WGS), more than 20 resistant elements and 5 resistant plasmids were observed. Notably, the tigecycline resistance of *K. pneumoniae* 1678 was not related to the plasmid-borne *tetA* gene but associated with the overexpression of AcrAB and OqxAB efflux pumps, according to the susceptibility results of *tetA*-transformant and the related mRNA quantification of RND efflux pumps. Except for tigecycline resistance, three plasmids, mediating resistance to colistin, Fosfomycin, and ceftazidime–avibactam, respectively, were focused. Detailed comparative genetic analysis showed that all these plasmids belonged to dominated epidemic plasmids, and harbored completed conjugation systems. Results of conjugation assay indicated that these three plasmids not only could transfer to *E. coli* J53 with high conjugation frequencies, respectively, but also could co-transfer to *E. coli* J53 effectively, which was additionally confirmed by the S1-PFGE plasmids profile. Moreover, multiple insertion sequences (IS) and transposons (Tn) were also found surrounding the vital resistant genes, which may form several novel mechanisms involved in the resistant determinants’ mobilization. Overall, we characterized and reported the uncommon co-existence and co-transferring of FosA3-, NDM-5, and MCR-1-encoding plasmids in a *K. pneumoniae* isolate, which may increase the risk of spread of these resistant phenotypes and needing great concern.

## Introduction

Carbapenem-resistant *Klebsiella pneumoniae* (CRKP) has recently emerged as a major class of bacterial pathogens that pose a significant threat to global public health, since it can cause high-fatal infections, and the treatment choices are very limited (Chen et al., 2014). The emergence of antibiotic resistance arises the development of several new antibiotics, such as tigecycline (Chopra, 2002) and ceftazidime–avibactam (Zhanel et al., 2013), and the re-evaluation of old antibiotics such as Fosfomycin and polymyxin as a potential regimen for treating such multidrug-resistant bacteria (Doi, 2019). Tigecycline, colistin, Fosfomycin, and ceftazidime–avibactam were considered as the most effective agents for the CRKP infection treatments, and were even regarded as the last “trump card” to defend against CRKP (Doi, 2019). However, clinical isolates resistant to those four antibiotics emerged frequently (Petrosillo et al., 2019; Fang et al., 2020; Yahav et al., 2020; Zurfluh et al., 2020; Yusuf et al., 2021). Hence, verifying the related mechanism and demonstrating the potential of the spread of these resistant phenotypes in clinical isolate are urgent, which are the vital clues to solve antibiotic resistance.

The acquisition of antibiotic resistance was always associated with mobile genetic elements (MGEs) such as conjugative and mobilizable plasmids and transposons (Partridge et al., 2018). In *Enterobacteriaceae*, Fosfomycin-modifying enzymes are the important factors to inactivate the Fosfomycin, genes encoding these enzymes (*fosA*) are frequently found on plasmids, transposons, or within integrons (Zurfluh et al., 2020). Lipopolysaccharide modifications are the key issues to reduce the antibiotic effect of polymyxin. In addition to the two-component systems (TCSs) *PhoP/PhoQ*, *PmrA/PmrB*, and *CrrA/CrrB* (Mcconville et al., 2020), the plasmid-mediated *mcr* genes (such as *mcr-1*) mediated enzymes are the most noteworthy way to modify lipopolysaccharides, which not only result in polymyxin resistance, but also result in the transferring of this antibiotic resistant-phenomena worldwide (Xiaomin et al., 2020). The tigecycline resistance is sometimes associated with the overexpression of the efflux pumps AcrAB and OqxAB (Bialek-Davenet et al., 2015). Meanwhile, mutations in tetracycline resistance factors, including efflux pumps (*tetA*, *tetB*, and *tetK*) (Foong et al., 2020; Xu et al., 2021) and other plasmid-borne tigecycline resistance genes, *tet(X)* (Sun et al., 2019) and *tmexCD-toprJ* (Hirabayashi et al., 2021), have also been reported to contribute to *K. pneumoniae* resistance to tigecycline. Although ceftazidime–avibactam (CAZ/AVI) exhibited remarkable inhibition to KPC carbapenemase, it is not active against Metallo-β-Lactamases (MBL)-producing bacteria, such as *bla*_*NDM*_-positive isolates (Yang et al., 2020). Overall, since almost all the antibiotic resistance could be carried by various MGEs, once the bacteria obtain multiple resistant elements simultaneously, they would become resistant to those antibiotic agents, and the therapeutic options would be very limited.

The co-occurrence of multiple resistant plasmids in one isolate often results in the resistance to almost all available antibiotics, and also promotes the dissemination of resistance determinants. Several studies also reported some co-existence of resistant-genes in *Enterobacteriaceae*, like *fosA3* and *bla*_*KPC–2*_ (Tang et al., 2020), or *mcr-1* and *bla*_NDM–5_ (Sun et al., 2016), these co-existences make the strain become extensively drug-resistant to multiple antibiotics. Notably, although there are several reports about the co-existence of *mcr-1* and *bla*_NDM–5_ in one plasmid or two separated plasmids, most of these plasmids were harbored by *E. coli* strains of animal origin or environmental origin, which is uncommon in *K. pneumoniae* (Yang et al., 2016; Quan et al., 2017; Mao et al., 2018; Wang et al., 2018, 2021; Chen et al., 2019; Liu and Song, 2019; Han et al., 2020; Yuan et al., 2021).

In this study, our aim was to characterize an XDR *K. pneumoniae* isolated from a clinical patient, which is not only highly resistant to carbapenems, but also resistant to all the alternative antibiotics, including tigecycline, ceftazidime–avibactam, Fosfomycin, and polymyxin. We applied whole-genome-sequencing (WGS) to explore the potential molecular mechanisms mediating this multidrug-resistance, and observed three key resistant plasmids. We also made a detailed analysis of the plasmid-backbone and the conjugation region to evaluate the potential movability, and applied the conjugation assay to further determine the dissemination risk of these resistant determinants. In addition to the plasmids, we described other related MEGs through the genetic comparisons as well. Overall, our goal was to report and describe a clinical multi-drug resistant *K. pneumoniae* clearly, and emphasize the possible risk of these strains.

## Materials and Methods

### Bacterial Strains

To explore the molecular epidemic feature of carbapenem-resistant *K. pneumoniae* in China mainland, we randomly collected 137 carbapenem-resistant *K. pneumoniae* isolates from blood samples of individual patients at nine hospitals in eight Chinese provinces, from January 2015 to December 2018. The isolates were cultivated with LB medium. We applied WGS to analyze the presence of resistance elements among these isolates, and observed *K. pneumoniae* strain 1678 co-harboring multiple resistance determinants including *fosA3*, *mcr-1*, and *bla*_NDM–5_, that were uncommon in other *K. pneumoniae. K. pneumoniae* strain 1678 was isolated from the blood samples of a 71-year-old patient in 2018, in a tertiary hospital in Shanghai, China. Plasmid transformation and conjugation were performed with *Escherichia coli* TOP10 and J53 (sodium-azide^R^) used as recipients for the selection of *tetA-, fosA3-, bla*_NDM–5_, or *mcr-1*-positive transformants and related transconjugants, respectively.

### Antimicrobial Susceptibility Test

The minimum inhibitory concentration (MIC) of the original isolate 1678 and all the transformants and transconjugants were determined by both broth microdilution and the polymyxin MIC was determined by the E-test methods following the Clinical and Laboratory Standards Institute guidelines. Briefly, for the broth microdilution and agar dilution method, pick 1–2 bacterial clones diluted with saline to 0.5McF, and then dilute such bacterial suspension to 0.5 × 10^–2^ McF with CAMHB broth. The cells were inoculated in prefabricated commercial 96-well antibiotic culture plates or antibiotic agars, 100 μL per well, and incubated overnight for 18 h at 37°C. E-test method using a colistin strip (concentration range, 0.016–256 μg/ml) (bioMérieux) was performed with Mueller–Hinton agar (MHA) (BD) plates in accordance with recommendations of the manufacturers. Notably, the Fosfomycin MIC was tested by the agar dilution using agar media supplemented with 25 μg/mL of glucose-6- phosphate. *Escherichia coli* ATCC25922 was used as a quality control strain for MIC determination. The interpretative breakpoints were based on CLSI2021 (Clinical and Laboratory Standards Clinical and Laboratory Standards Institute, 2021a; Clinical and Laboratory Standards Clinical and Laboratory Standards Institute, 2021b).

### Quantitation of mRNA Expression

To explore whether the tigecycline-resistant phenotype was related with the overexpression of AcrAB and OqxAB efflux pumps, we applied q-RT-PCR (quantitative-real-time-PCR) to measure the relative gene expression. All the primers were listed in Supplementary Table 1. RNA manipulation and real-time PCR were performed as described previously (Zheng et al., 2018). All bacterial samples were cultured in LB medium that did not contain any antibiotics. RNA was isolated as per the protocol of the MiniBEST Universal RNA extraction kit (TaKaRa, Tokyo, Japan). RNA samples for real-time PCR were pre-treated with DNase I (TaKaRa, Tokyo, Japan). Real-time PCR was conducted on a 7,500 system (Applied Biosystems, Foster City, CA, United States) using SYBR Premix ExTag (Takara, Tokyo, Japan). The expression of target genes was standardized relative to the 16S rRNA housekeeping gene *rrsE*. The expression levels of the target genes were compared with those of *K. pneumoniae* ATCC 13,883 (tigecycline susceptible). The relative expression levels of genes were calculated using the ΔΔ*CT* method. All assays were performed in triplicate with three independent RNA preparations.

### Whole Genome Sequencing and Bioinformatics Analysis

The genomic DNA of 1678 was extracted using a commercial DNA extraction kit (Qiagen, Germany) and was sequenced using short- and long-read massively parallel sequencing. The paired-end short Illumina reads were used to correct the long PacBio reads utilizing *proovread*, and then the corrected PacBio reads were assembled *de novo* utilizing *SMARTdenovo^1^*. Resistant plasmid replicons were identified using the PlasmidFinder database using the minimum coverage and minimum identities of 90%^2^. Acquired antibiotic resistance genes were identified using ResFinder^3^ with the default threshold. To determine whether the plasmids could self-transmission, we used the oriTfinder^4^ to conduct a detailed analysis of the conjugation module, including the origin of transfer site (*oriT*), relaxase gene, type IV coupling protein (T4CP) gene, and the type IV secretion system gene cluster (T4SS). The related insertion sequences (IS) and transposons (Tn) were determined through the ISFinder^5^. BLAST Ring Image Generator (BRIG) was used to compare key resistant plasmids with other representative plasmids to further generate circular plasmid maps. Easyfig software was used to generate comparison of gene environment surrounding the vital resistant genes.

### Transformation Assay

In order to test whether these plasmids could mediate the corresponding resistant phenotype, we extracted and transformed each single resistant plasmid to *E. coli* Top 10, and then tested the antibiotic susceptibility of all transformants. The plasmid extraction and transformation processes were performed as previously described (Yin et al., 2017).

We used the phenol-chloroform extraction method to extract the plasmids in 1678. Then, we mixed 4 μl extracted plasmids and *E. coli* Top 10 competent cells together, placed it on ice for 30 min, put it in a 42°C water bath for 90 s, and then took it out and placed it on ice for 2 min. After that, we used LB broth to resuscitate the strain, and screened the transformants on appropriate antibiotic plates. Successful transformants were determined by PCR. All the transformants were selected in appropriate antibiotics [Amp, 100 mg/L (*bla*_NDM–5_); Fosfomycin, 16 mg/L (*fosA3*); colistin, 4 mg/L (*mcr-1*); tetracycline, 30 mg/L (*tetA*)].

### Conjugation Assay

We applied conjugation assay (Zhou et al., 2020) to evaluate whether these resistant plasmids could be transferred or co-transferred from *K. pneumoniae* 1678 (donor isolate) to *E. coli* J53 (recipient isolate). The donors and recipients were cultured to the logarithmic phase, mixed in 1:1 ratio, centrifuged at 8,000 × *g* for 1 min, and then resuspended in 20 μl MgSO_4_ (10 mM). The resuspension was spotted on the Luria Bertani (LB) plate and incubated at 37°C overnight. Subsequently, the serial dilutions were plated in media with appropriate antibiotics [Amp, 100 mg/L (*bla*_NDM–5_); Fosfomycin, 16 mg/L (*fosA3*); colistin, 4 mg/L (*mcr-1*); sodium azide, 100 mg/L (J53 recipient)]. The conjugation frequency was calculated as the number of transconjugants per donor. All transconjugants were confirmed by PCR for the presence of *fosA3*, *bla*_NDM–5_, and *mcr-1* genes. All the primers were listed in Supplementary Table 1.

### S1-Pulsed-Field Gel Electrophoresis Assay

The S1-pulsed-field gel electrophoresis (S1-PFGE) was performed to further determine the existence of plasmids in the original isolate *K. pneumoniae* 1678, and its transformants and transconjugants. PFGE plugs of all these strains were prepared and digested as previously described (Ai et al., 2021). Briefly, the isolates were embedded in 10 g/L of Seakem Gold gel, and digested with endonuclease S1 nuclease (Takara, Dalian, China). PFGE analysis was performed with a CHEF mapper system (Bio-Rad). The digested DNA fragments were separated for 19 h at 6 V/cm, 14°C on a 1.0% agarose gel (Bio-Rad) with pulse times of 4–40 s. *XbaI*-digested Salmonella H9812 DNA was used as the DNA marker. The nucleic acid dye Gel-red (Yeasen, China) was used to stain the DNA in the gels.

### Nucleotide Sequence Accession Numbers of *Klebsiella pneumoniae* 1678

The complete nucleotide sequences of the chromosome and plasmids p1678-2, p1678-3, p1678-4, p1678-5, and p187–6 were submitted to GenBank under accession numbers CP080445, CP080446, CP080447, CP080448, CP080449, and CP080450, respectively.

## Results

### *Klebsiella pneumoniae* 1678 Was a Typical Extensively Drug-Resistant Isolate

In order to clarify the antibiotic-resistant phenotype of *Klebsiella pneumoniae* 1678, we tested the susceptibility of 26 antibiotics in this strain (Table 1), especially including Fosfomycin, tigecycline, colistin, and ceftazidime–avibactam that were known for their robust bactericidal effect against CRKP. Our results indicated the *K. pneumoniae* 1678 was a representative multi-drug resistant strain, which not only exhibited high-level resistance to all β-lactam antibiotics and carbapenems, but was even resistant to tigecycline, Fosfomycin, colistin, and ceftazidime–avibactam (Table 1). These resistance profiles indicated the treatment option for the infection caused by *K. pneumoniae* 1678 would be limited.

**TABLE 1:** Antimicrobial drug susceptibility profiles.

Antibiotics	MIC (mg/L)/antimicrobial susceptibility												
				Transformants				Transconjugants					
	1678	Top10	J53	p1678–6-TOP10 (TetA)	p1678-3 Top10 (FosA3)	p1678-4-Top10 (NDM-5)	p1678-5-Top10 (MCR-1)	p1678-3-J53 (FosA3)	p1678-4-J53 (NDM-5)	p1678-5-J53 (MCR-1)	p1678-3 and 5-J53 (FosA3+MCR-1)	p1678-4 and 5-J53 (NDM-5+MCR-1)	p1678-3 and 4 and 5-J53 (FosA3+NDM-5+MCR-1)
**MEM**	**>16/R**	≤0.06/S	≤0.06/S	≤0.06/S	≤0.06/S	**16/R**	≤0.06/S	≤0.06/S	**16/R**	≤0.06/S	≤0.06/S	**16/R**	**16/R**
**IPM**	**16/R**	≤0.25/S	≤0.25/S	≤0.25/S	≤0.25/S	**4/R**	≤0.25/S	≤0.25/S	**4/R**	≤0.25/S	≤0.25/S	**8/R**	**8/R**
**ETP**	**>2/R**	≤0.015/S	≤0.015/S	≤0.015/S	≤0.015/S	**>2/R**	≤0.015/S	≤0.015/S	**>2/R**	≤0.015/S	≤0.015/S	**>2/R**	**>2/R**
**Caz/AVI**	**>16/4/R**	≤0.5/4/S	≤0.5/4/S	≤0.5/4/S	≤0.5/4/S	**>16/4/R**	≤0.5/4/S	≤0.5/4/S	**>16/4/R**	≤0.5/4/S	≤0.5/4/S	**>16/4/R**	**>16/4/R**
**TGC**	**8/R**	≤0.25/S	≤0.25/S	≤**0.25/S**	≤0.25/S	≤0.25/S	≤0.25/S	≤0.25/S	≤0.25/S	≤0.25/S	≤0.25/S	≤0.25/S	≤0.25/S
**POL**	**16/R**	0.25/S	0.25/S	0.25/S	0.25/S	0.25/S	**16/R**	0.25/S	0.25/S	**16/R**	**16/R**	**16/R**	**16/R**
**FOS**	**>256/R**	0.25/S	0.25/S	0.25/S	**>256/R**	0.25/S	0.25/S	**>256/R**	0.25/S	0.25/S	**>256/R**	0.25/S	**>256/R**
AMP	**>32/R**	≤8/S	≤8/S	≤8/S	**>32/R**	**>32/R**	≤8/S	**>32/R**	**>32/R**	≤8/S	**>32/R**	**>32/R**	**>32/R**
CZO	**>32/R**	≤2/S	≤2/S	≤2/S	**>32/R**	**>32/R**	≤2/S	**>32/R**	**>32/R**	≤2/S	**>32/R**	**>32/R**	**>32/R**
CAZ	**>128/R**	≤0.25/S	≤0.25/S	≤0.25/S	**>128/R**	**>128/R**	≤0.25/S	**>128/R**	**>128/R**	≤0.25/S	**>128/R**	**>128/R**	**>128/R**
FEP	**>16/R**	≤0.5/S	≤0.5/S	≤0.5/S	≤0.5/S	**>16/R**	≤0.5/S	≤0.5/S	**>16/R**	≤0.5/S	≤0.5/S	**>16/R**	**>16/R**
CSL	**>64/32/R**	≤16/8/S	≤16/8/S	≤16/8/S	≤16/8/S	**>64/32/R**	≤16/8/S	≤16/8/S	**>64/32/R**	≤16/8/S	≤16/8/S	**>64/32/R**	**>64/32/R**
SAM	**>32/16/R**	≤16/4/S	≤16/4/S	≤16/4/S	≤16/4/S	**>32/16/R**	≤16/4/S	≤16/4/S	**>32/16/R**	≤16/4/S	≤16/4/S	**>32/16/R**	**>32/16/R**
FOX	**>32/R**	≤8/S	≤8/S	≤8/S	≤8/S	**>32/R**	≤8/S	≤8/S	**>32/R**	≤8/S	≤8/S	**>32/R**	**>32/R**
CXM	**>16/R**	8/S	8/S	8/S	**>16/R**	**>16/R**	8/S	**>16/R**	**>16/R**	8/S	**>16/R**	**>16/R**	**>16/R**
CTX	**>64/R**	≤0.12/S	≤0.12/S	≤0.12/S	**64/R**	**>64/R**	≤0.12/S	**64/R**	**>64/R**	≤0.12/S	**64/R**	**>64/R**	**>64/R**
TZP	**>128/4/R**	≤16/4/S	≤16/4/S	≤16/4/S	≤16/4/S	**>128/4/R**	≤16/4/S	≤16/4/S	**>128/4/R**	≤16/4/S	≤16/4/S	**>128/4/R**	**>128/4/R**
AMC	**>32/16/R**	≤8/4/S	≤8/4/S	≤8/4/S	≤8/4/S	**>32/16/R**	≤8/4/S	≤8/4/S	**>32/16/R**	≤8/4/S	≤8/4/S	**>32/16/R**	**>32/16/R**
LVX	**>8/R**	≤0.12/S	≤0.12/S	≤0.12/S	≤0.12/S	≤0.12/S	≤0.12/S	≤0.12/S	≤0.12/S	≤0.12/S	≤0.12/S	≤0.12/S	≤0.12/S
MFX	**>2/R**	≤0.25/S	≤0.25/S	≤0.25/S	≤0.25/S	≤0.25/S	≤0.25/S	≤0.25/S	≤0.25/S	≤0.25/S	≤0.25/S	≤0.25/S	≤0.25/S
TCY	**>16/R**	≤2/S	≤2/S	**16/R**	≤2/S	≤2/S	≤2/S	≤2/S	≤2/S	≤2/S	≤2/S	≤2/S	≤2/S
GEN	**16/R**	≤1/S	≤1/S	≤1/S	≤1/S	≤1/S	≤1/S	≤1/S	≤1/S	≤1/S	≤1/S	≤1/S	≤1/S
AMK	≤16/S	≤16/S	≤16/S	≤16/S	≤16/S	≤16/S	≤16/S	≤16/S	≤16/S	≤16/S	≤16/S	≤16/S	≤16/S
ATM	**>16/R**	≤4/S	≤4/S	≤4/S	**>16/R**	≤4/S	≤4/S	**>16/R**	≤4/S	≤4/S	≤4/S	≤4/S	**>16/R**
NIT	**64/I**	≤16/S	≤16/S	≤16/S	≤16/S	≤16/S	≤16/S	≤16/S	≤16/S	≤16/S	≤16/S	≤16/S	≤16/S
SXT	**>4/76/R**	≤0.5/9.5/S	≤0.5/9.5/S	≤0.5/9.5/S	≤0.5/9.5/S	≤0.5/9.5/S	≤0.5/9.5/S	≤0.5/9.5/S	≤0.5/9.5/S	≤0.5/9.5/S	≤0.5/9.5/S	≤0.5/9.5/S	≤0.5/9.5/S

*MEM, Meropenem; IPM, Imipenem; ETP, Ertapenem; Caz/AVI, ceftazidime–avibactam; TGC, Tigecycline; POL, Polymixin B; FOS, Fosfomycin; AMP, Ampicillin; CZO, Cefazolin; CAZ, Ceftazidime; FEP, Cefepime; CSL, Cefoperazone/Sulbactam; SAM, Ampicillin/Sulbactam; FOX, Cefoxitin; CXM, Cefuroxime; CTX, Cefotaxime; TZP, Piperacillin/Tazobactam; AMC, Amoxicillin/Clavulanic acid; LVX, Levofloxacin; MFX, Moxifloxacin; TCY, Tetracycline; GEN, Gentamicin; AMK, Amikacin; ATM, Aztreonam; NIT, Nitrofurantoin; SXT, Trimethoprim/Sulfamethoxazole. The other resistance phenotype like LVX, MFX, GEN, NIT, or SXT resistance did not present in these transformants or transconjugants in table because these elements are located on p1678-2 plasmid, not p16783-3(fosA3), p1678-4(bla_NDM–5_), p1678-5(mcr-1), and p1678-6(tetA). The bold values indicated important resistance genes and resistance phenotypes.*

### *Klebsiella pneumoniae* 1678 Co-harboring *fosA3*, *bla*_NDM–5_, and *mcr-1*

**FIGURE 1:**
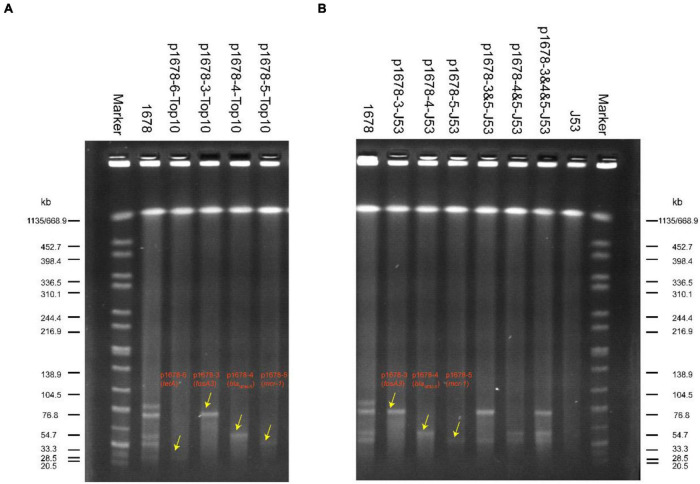
SI-PFGE profiles of original *K. pneumoniae* 1678 and its transformants **(A)** and transconjugants **(B)**. Lane marker was *XbaI*-digested DNA of Salmonella Braenderup H9812; Lane 1678 and Lane J53 were used as positive reference and negative control, respectively; transformants: p1678-6-Top10 (*tetA*), p1678-3-Top10 (*fosA3*), p1678-4-Top10(*bla*_NDM–5_), and p1678-5-Top10(*mcr-1*). Transconjugants: p1678-3-J53(*fosA3*), p1678-4-J53(*bla*_NDM–5_), p1678-5-J53(*mcr-1*), p1678-3 and 5-J53 (*fosA3* and *mcr-1*), p1678-4 and 5-J53 (*bla*_NDM–5_and *mcr-1*), and p1678-3 and 4 and 5-J53 (*fosA3*, *bla*_NDM–5_, and *mcr-1*).

To further investigate the related mechanism that mediated the extensively drug-resistant resistant (XDR) characteristic of *K. pneumoniae* 1678, we used WGS to deeply describe the genomic information of the XDR bacteria. According to the MLST analysis, the *K. pneumoniae* 1678 was typed as ST485. We found more than 20 resistant elements and 5 resistant plasmids in this isolate (Table 2). Moreover, three key resistance genes were focused, which played a significant role in the formation of resistance to Fosfomycin (*fosA3*), carbapenems, ceftazidime–avibactam (*bla*_NDM–5_), and colistin (*mcr-1*). In addition to the molecular detection of these crucial resistant elements, we also extracted and transformed each resistant plasmid to *E. coli* Top 10 (Figure 1A) and tested whether these plasmids could mediate the corresponding resistant phenotype. Antibiotic susceptibility results of all transformants well proved the role of the resistant plasmids (Table 2).

**TABLE 2:** General features, antimicrobial resistance genes of plasmids in *K. pneumoniae* 1678.

Characteristics	Results				
	p1678-2	p1678-3	p1678-4	p1678-5	p1678-6
Accension number	CP080446	CP080447	CP080448	CP080449	CP080450
Length(bp)	90,943	76,526	46,161	33,309	24,774
GC content (%)	54	52	47	42	54
No. of ORF	116	92	59	42	29
Incompatibility group	IncFIIK(IncQ1	**IncFII**	**IncX3**	**IncX4**	IncR
Conjugal ability	No	**Yes**	**Yes**	**Yes**	No
Resistant genes					
	*bla*_OXA–1_	***fosA3***	***bla*_NDM–5_**	***mcr-1***	*TetA*
	aac(3)-Iid	*bla*_CTX–M–55_			
	aph(3′)-Ia	*bla*_TEM–141_			
	sul2, sul1				
	sul2, sul1;				
	aac(6′)-Ib-cr				
	aph(3′)-Ia				
	aac(3)-Iid				
	mph(A)				
	aadA16				
	qnrB52				
	ARR-3				
	catB3				

*The bold values indicated important resistance genes and resistance phenotypes.*

### Tigecycline Resistance Was Mediated by the Overexpression of RND-Type Efflux Transporters

The mechanisms underlying tigecycline resistance are complex. Previous studies demonstrated the mutation of plasmid-borne *tet(A)* could be an important factor causing tigecycline resistance in *K. pneumoniae.* Accordingly, we compared the amino-acid sequence of the Tet(A) protein of *K. pneumoniae* 1678 and the mutated Tet(A) confirmed before (Supplementary Figure 1). We found the Tet(A) carried by *K. pneumoniae* 1678 owned the same mutated characteristic which Xu (Xu et al., 2021) described (Supplementary Figures 1, 3). Moreover, we also detected the mRNA expression of the *tet(A)* gene in the *E. coli* transformants to ensure the *tet(A)* gene could be expressed normally (Supplementary Figure 2). However, we could not detect the tigecycline resistant phenotype, but only the resistant to tetracycline in the *tet(A)-*transformants (Table 2), which indicated that this mutation may not contribute to tigecycline resistance in *K. pneumoniae* 1678.

**FIGURE 2:**
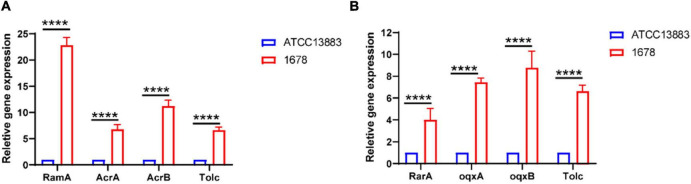
Overexpression of AcrAB and OqxAB in clinical *K. pneumoniae* 1678 isolate. The expression levels of AcrA/B **(A)**, OqxA/B **(B)**, and related transcriptional regulators was determined by qRT-PCR. The *K. pneumoniae* ATCC13883 is used as the reference strain (expression = 1.0). The data represent the mean standard deviation for three independent biological replicates. Differences between different strains, regarding related gene expression, were statistically analyzed using a two-tailed Student’s *t*-test with Bonferroni correction. ****p < 0.0001.

In addition to the mutation of Tet(A) protein, the overexpression of the RND-type efflux pumps AcrAB and OqxAB has been shown to play a crucial role in tigecycline resistance in *K. pneumoniae* (Bialek-Davenet et al., 2015; Li et al., 2017). Our qRT-PCR experiments indicated that *K. pneumoniae* 1678 overexpressed the AcrAB–TolC pathway genes *acrA/B* and *tolC* (>sixfold greater than the tigecycline susceptible *K. pneumoniae* ATCC 13883 reference strain) (Figure 2A), and was also observed to overexpress *oqxA* and *oqxB* (range 6.432- to 10.435-fold compared with the reference strain levels) (Figure 2B). Moreover, the activating regulator of AcrAB (*ramA*) and OqxAB (*rarA*) also exhibited the same expression level (Figure 2). What is more, the mutation in RamR protein was also analyzed, for it is the negative regulator of RamA (Zheng et al., 2018), and we found an amino acid mutation (L44M) compared with the reference sequence (Accension number: ADI49705.1), but the phenotype is unproved. We assumed this may do a potential favor for the overexpression of AcrAB. These results showed the overexpression of RND-type efflux transporters contributed to the tigecycline resistance of *K. pneumoniae* 1678.

### Comparative Genomics of the Plasmids Carrying Resistance Genes

**FIGURE 3:**
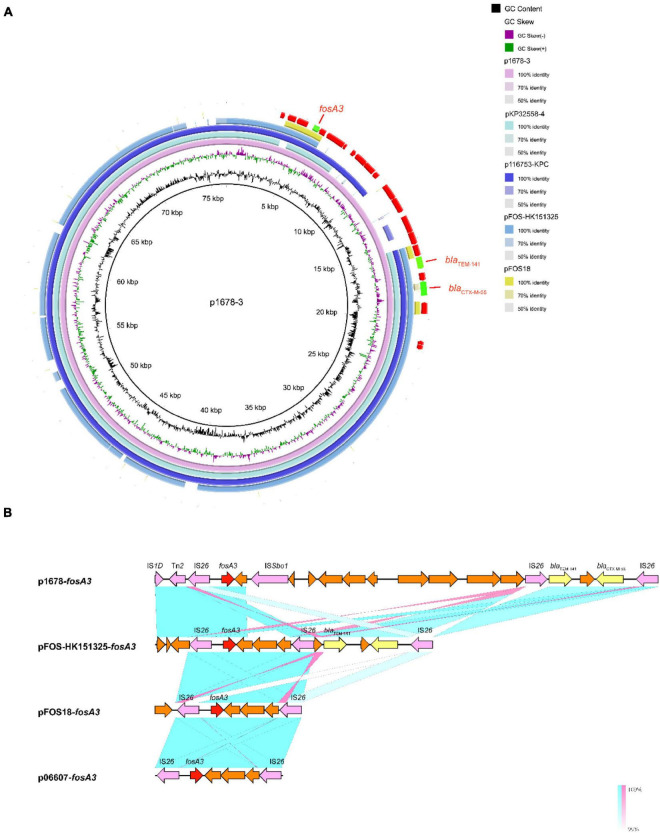
Comparative analysis of pl687-3 plasmids with other reference plasmids. **(A)** p1678-3 (CP080447) was used as the reference plasmid to perform genome alignment with pFOS-HK151325 (JX627737, first *fosA3* plasmid from a clinical *E. coli* identified in China) and pFOS18 (KJ653815, first *fosA3* plasmid from a clinical *K. pneumoniae* identified in China). Moreover, p1678-3 was also compared with another two similar plasmids pKP32558-4 (CP076034.1, *K. pneumoniae*) and p116753-KPC (MN891682.1, *K. pneumoniae*). The red arrows represent CDs. **(B)** Linear comparison of the *fosA3* region. The *fosA3* region was compared with the regions extracted from pFOS-HK151325, pFOS18, and p06607 (AB522970, first *fosA3* plasmid).

We have confirmed that the key resistant genes were all located on plasmids. As plasmids are often transmissible between bacteria, and some have spread globally, we made detailed analysis of these resistant plasmids, aiming to further clarify the resistance mechanism and potential dissemination threats of *K. pneumoniae* 1678. p1678-3 was a typical IncFII-type plasmid, harboring a completed conjugation system, and shared 81% identity with pFOS-HK151325, the first *fosA3* plasmid from a clinical *E. coli* identified in China (Figure 3A). Moreover, p1678-3 was also highly similar to pKP32558-4 (89% identity, CP076034.1, *K. pneumoniae*) and p116753-KPC (95% identity, MN891682.1, *K. pneumoniae*). The genetic differences between p1678-3 and these plasmids were most concentrated in the surrounding genes of *fosA3* gene, which may be related to the mobility insertion of IS elements (Figure 3A).

**FIGURE 4:**
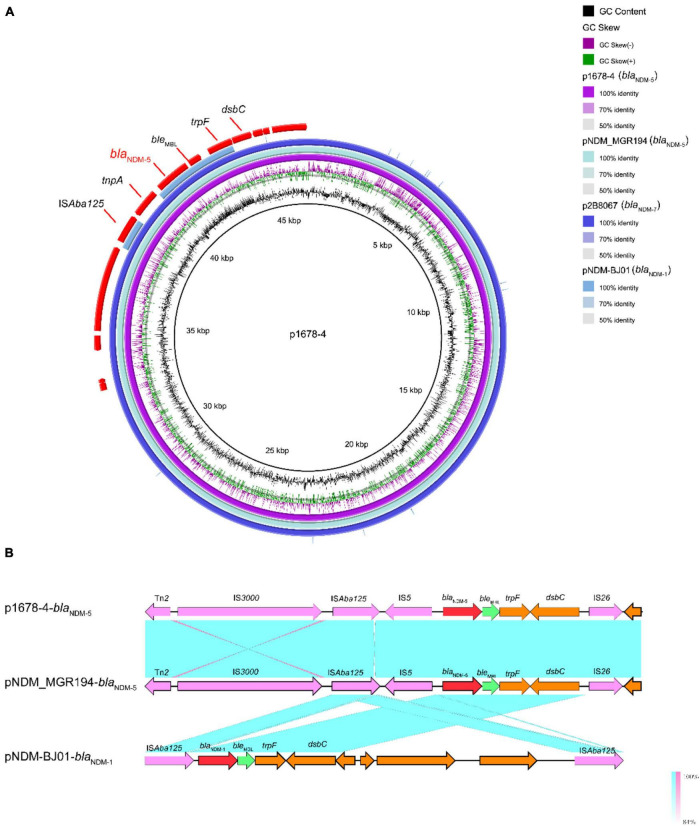
Comparative analysis of pl687-4 plasmids with other reference plasmids. **(A)** p1678-4 (CP080448) was used as the reference plasmid to perform genome alignment with pNDM-MGR194 (*bla*_NDM–5_, KF220657.1), p2B8067 (*bla*_NDM–7_, CP070442.1), and pNDM-BJ01 (*bla*_NDM–1_, JQ001791). The red arrows represent CDs. **(B)** Linear comparison of the *bla*_NDM–5_ region. The *bla*_NDM–5_ region was compared with the *bla*_NDM_ regions extracted from pNDM-MGR194 (*bla*_NDM–5_ reference plasmid) and pNDM-BJ01 (classical *bla*_NDM_-Tn*125* transposon).

The wide dissemination of *bla*_NDM_ genes is largely mediated by certain plasmids, particularly those of the IncX3 type, which p1678-4 plasmid belonged to. Moreover, the genetic context of the p1678-4 plasmid (Figure 4A) was nearly identical to that of the human *K. pneumoniae* plasmid pNDM-MGR194 (2015, IncX3, *bla*_NDM–5_, KF220657.1) previously reported in India (Krishnaraju et al., 2015) and was also highly similar to p2B8067 (2021, IncX3, *bla*_NDM–7_, CP070442.1). These results indicated that no matter the variants of *bla*_NDM_, the IncX3 plasmid was a major vehicle in mediating the dissemination of *bla*_NDM_. Similar to the IncFII plasmids described before (Bi et al., 2018), IncX3 plasmid also could be self-transferred among *Enterobacteriaceae*, supported with the results of the conjugation mode analysis of p1678-4 plasmid (Table 2).

**FIGURE 5:**
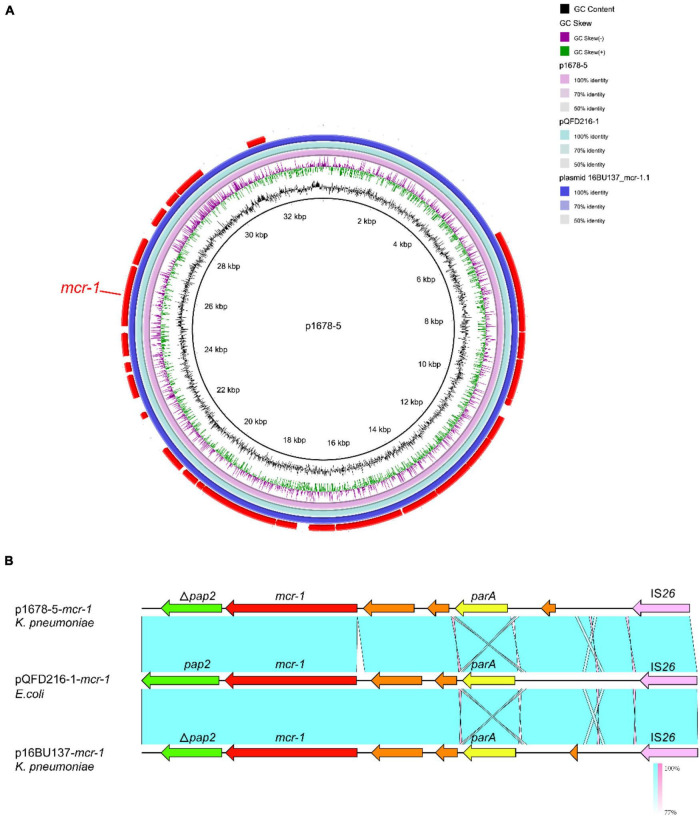
Comparative analysis of pl687-5 plasmids with other reference plasmids. **(A)** p1678-5 (CP080449) was used as the reference plasmid to perform genome alignment with pQDFD216-1 (CP053212.1, *E. coli*) and plasmid 16BU137 (MT316509.1, *K. pneumoniae*). The red arrows represent CDs. **(B)** Linear comparison of the *mcr-1* region. The *mcr-1* region was compared with the *mcr-1* regions extracted from pQDFD216-1 and pQDFD216-1.

p1678-5 plasmid was a 33,309-bp circular molecule with *repA* belonging to IncX4, harboring *mcr-1* resistance element (Figure 5A). Previous studies have demonstrated most plasmids carrying *mcr-1* are transferable, and IncX4 was dominant *mcr-1*-carrying plasmid types (Xiaomin et al., 2020). According to the genomic comparation, we found p1678-5 was almost identical to both pQDFD216-1 (CP053212.1) plasmid identified in *E. coli* and plasmid 16BU137 (MT316509.1) from *K. pneumoniae.* These results showed IncX4 plasmids harboring *mcr-1* could disseminate in different species of *Enterobacteriaceae*, and the completed conjugative element also can be found in p1678-5 plasmid (Table 2). In addition, we also make a comparison between p1678-5 and the first *mcr-1* plasmid pHNSHP45 (NZ_KP347127.1, IncI2) (Liu et al., 2016), and found low identity between two plasmids (Supplementary Figure 4).

### Resistant Plasmids Could Be High-Efficient Self-Transferred or Co-transferred

We have known these three resistance plasmids were all bioinformatic predicted to carry essential conjugative modules (*oriT*, Relaxase, T4CP, and T4SS) and most of these types of plasmids have proved to be movable (Partridge et al., 2018). However, it is not common for such three plasmids to co-exist in one *K. pneumoniae*, and the transferring and co-transferring pattern was unclear. Here, we applied conjugation assay to imitate and evaluate the dissemination ability of these three plasmids in *K. pneumoniae* 1678. We found all these three plasmids could transfer to *E. coli* J53 with high conjugation frequencies (1.42 × 10^–4^ − 7.9 × 10^–3^), especially for the *mcr-1* plasmid (p1678-5) (Table 3). In addition to the self-transferring of a single plasmid, we also observed co-transfer of two plasmids and even three plasmids and the co-conjugation frequencies of two plasmids only decrease 1-log compared to a single plasmid (Table 3). Although the co-conjugation frequencies of three plasmids was low, the potential clinical threat could not be ignored, since the clonal spread will accelerate the spread of these resistance genes. Moreover, the plasmid pattern of S1-PFGE further proved the transferring profile of the *K. pneumoniae* 1678 (Figure 1B), and the antibiotic MICs of these transconjugants also confirmed the spread of resistance phenotype of *K. pneumoniae* 1678 (Table 1).

**TABLE 3:** Conjugation frequency of resistant plasmids identified in *K. pneumoniae* 1678.

Plasmid	Resistance gene	No. of independent determinations	Conjugation frequencies	
			Mean	Range
p1678-3	*fosA3*	3	2.25 × 10^–4^	1.97 × 10^–4^ − 2.75 × 10^–4^
p1678-4	*bla*_NDM–5_	3	1.84 × 10^–4^	1.42 × 10^–4^ − 2.37 × 10^–4^
p1678-5	*mcr-1*	3	5.41 × 10^–3^	2.94 × 10^–3^ − 7.9 × 10^–3^
Co-transfer of plasmids				
Co-transfer of p1678-3 and p1678-5	*fosA3* + *mcr-1*	3	1.78 × 10^–5^	1.29 × 10^–5^ − 2.77 × 10^–5^
Co-transfer of p1678-4 and p1678-5	*bla*_NDM–5_ + *mcr-1*	3	2.25 × 10^–5^	1.62 × 10^–5^ − 2.92 × 10^–5^
Co-transfer of p1678-3 and p1678-4 and p1678-5	*fosA3* + *bla*_NDM–5_ + *mcr-1*	3	9.63 × 10^–8^	8.08 × 10^–8^− 10.38 × 10^–8^

### Mobile Genetic Elements Associated With *fosA3*, *bla*_NDM–5_, and *mcr-1*

The capture, accumulation, and dissemination of resistance genes are not only due to the spread of plasmids, but also to the actions of other MGEs, such as IS and Tn. To comprehensively evaluate the dissemination potential of these resistance genes in *K. pneumoniae* 1678, we also analyze the MGEs surrounding them. IS*26* seems to be the key element in the mobilization of *fosA3*, since it not only composes a composite transposon surrounding *fosA3* in the p1678-3 plasmids, but also surrounding the *fosA3* of pFOS-HK151325 plasmid (Yang et al., 2019) (2013, JX627737, *E. coli*, China), of pFOS18 plasmid (Yang et al., 2019) (2015, KJ653815, *K. pneumoniae*, China), and of p06607 plasmid (Yang et al., 2019) (2010, AB522970, first *fosA3* plasmid emerged in the world). Moreover, the same IS*26*-composite transposons were also observed to frequently contain additionally a *bla*_CTX–M_ gene. Compared to the first *fosA3* plasmid, there are more than 10 ORFs inserted around *fosA3* in p1678-3 plasmid, with some new IS, some elements associated with transcriptional regulation, and other resistance elements (Figure 3B).

The genetic contexts of *bla*_NDM_ share two common features. The insertion sequence IS*Aba125* (intact or truncated) is always upstream of *bla*_NDM_, while a bleomycin resistance gene, *ble*_MBL_, is always downstream. Further downstream of *ble*_MBL_, there are usually located *trpF* and *dsbC* genes (Figure 4B). Although the *bla*_NDM_-1 in *Acinetobacter* spp. is located within IS*Aba125*-based composite transposon Tn*125*(pNDM-BJ01, JQ001791) (Wu et al., 2019), it was always interrupted or truncated in *Enterobacteriaceae*. In p1678-4, Tn*125* was truncated by IS*5*, IS*26*, and IS*300* (Figure 4B). These new genetic contexts in p1678-4 may form a new mechanism involved in the mobilization of *bla*_NDM–5_.

Similar to other *mcr-1*-carrying IncX4-type plasmids (Xiaomin et al., 2020), the typical IS*26-parA-mcr-1.1-pap2* cassette was identified in p1678-5, with *pap2* gene partitional truncated (Figure 5B). Although IS*Apl1* has been described as the most common IS element adjacent to *mcr-1* at one or both ends, we did not observe it surrounding the *mcr-1* gene.

## Discussion

Extensively drug-resistant *K. pneumoniae* constitutes the major sources of nosocomial infections with extraordinary drug resistance. The prevalent resistance plasmids are responsible for the sudden increase in the population of multidrug resistance among *K. pneumoniae* isolates (Tzouvelekis et al., 2012; Chen et al., 2014). In this study, we described a multi-drug resistant *K. pneumoniae* 1678 co-harboring three self-transmissible resistant plasmids, which mediated the resistance for carbapenems, Fosfomycin, colistin, and ceftazidime–avibactam. The co-existing of these plasmids not only conferred the multi-drug resistant phenotype to *K. pneumoniae* 1678, but also held the potential threat to co-transfer to other isolates.

Tigecycline has been considered as an effective antibiotic against CRKP *in vitro*, and is also considered as one of the last-resort antibiotics against CRKP infections (Chopra, 2002; Doi, 2019). Unfortunately, tigecycline resistance has been reported frequently in clinics (Fang et al., 2020). In this study, we found *K. pneumoniae* 1678 was resistant to tigecycline. We identified the *tet(A)* gene in this isolate, and also confirmed the mutations on Tet(A) protein (Supplementary Figure 1). Several studies had verified the plasmid-borne mutated *tet(A)* gene or the synergy of TetA and RND-type efflux transporters play an important role in causing tigecycline resistance (Bialek-Davenet et al., 2015; Foong et al., 2020; Xu et al., 2021). However, we found the p1678-6 plasmid, harboring mutated *tet(A)* gene, could not result in tigecycline resistance. According to the genetic comparison of p1678-6 plasmid with other *tet(A)*-plasmid (tigecycline-resistant), we found p1678-6 plasmid shared low identity with them. Further to analyze the genetic components surrounding *tet(A)* gene, we found the IS and Tn elements in p1678-6 plasmid also different. As the IS elements sometimes would affect promoter activity (Partridge et al., 2018), we assumed the expression discrepancy of *tet(A)* gene in p1687-6 plasmid with other tigecycline-resistant-*tet(A)* plasmids may be accounted for in this antibiotic-susceptibility phenomenon. Although the Tet(A) protein did not contribute to the tigecycline resistance, we observed the overexpression of the efflux pumps AcrAB and OqxAB in *K. pneumoniae* 1678, another key factor mediating the tigecycline resistance (Bialek-Davenet et al., 2015; Li et al., 2017). Hence, in this study, the overexpression of RND-type efflux pumps played a crucial role in tigecycline resistance in *K. pneumoniae* 1678.

Except for resistant to tigecycline, the resistant to Fosfomycin, colistin, carbapenems, and ceftazidime–avibactam in *K. pneumoniae* 1678 were all associated with the typical resistant plasmids. Both *fosA3* and *bla*_CTX–M–55_ gene were located on p1678-3 plasmid, a typical IncFII plasmid. Previous studies have demonstrated that Fosfomycin-modifying enzymes were present on plasmids belonging to the IncF, IncN, IncA/C, IncHI2, and IncX1 family, whereas IncF was the predominant plasmid incompatibility type (Yang et al., 2019; Zurfluh et al., 2020). Although there existed several FosA variants, FosA3 was the most frequently found Fosfomycin-modifying enzyme worldwide, and many studies have confirmed the dissemination of the *fosA3* gene is closely associated with that of the ESBL gene *bla*_CTX–M_ (Yang et al., 2019), which was consistent with the findings in the p1678-3 plasmid. Our results also showed the p1678-3 plasmid had the completed conservative 35-kb conjugation module of IncF plasmids (Bi et al., 2018), and the self-transmissibility of this plasmid was also confirmed through conjugation assay. As we know, Fosfomycin was a potential regimen for treating extensively drug-resistant bacteria especially for carbapenemase-producing *Enterobacteriaceae* (CRE), once these isolates uptake the plasmids like p1678-3, the infection treatment would become limited.

*bla*_NDM–5_ plasmid (p1678-4) and *mcr-1* plasmid (p1678-5) all belong to IncX-type plasmid, holding a smaller size than IncF plasmid, which sometimes makes it easier for movement. p1678-4 plasmid belongs to IncX3 type, the major vehicle in mediating the dissemination of *bla*_NDM_ (Wu et al., 2019). Several NDM variants have been reported, which commonly contain between 1 and 5 amino acid substitutions compared to NDM-1. Notably, NDM-5 variant, containing the V88L substitution has repeatedly been reported to exhibit enhanced carbapenemase activity (Wu et al., 2019). MICs of ertapenem against strains producing NDM-5 were 4- or 8-fold higher than those against strains producing NDM-1 (Wu et al., 2019). Moreover, the novel antibiotic agent ceftazidime–avibactam used alone also makes no defense against NDM-5 carbapenemase (Yang et al., 2020). All this information indicated that the spread and pandemic of such IncX3-type p1678-4 plasmid could pose a huge risk to public health.

Like tigecycline, Fosfomycin, and ceftazidime–avibactam, colistin also is a robust antibiotic against infections caused by CRE (Doi, 2019). However, CRKP 1678 also harbored the pandemic IncX4 *mcr-1* plasmid (p1678-5), which conferred resistance to colistin. Most plasmids carrying *mcr-1* are reported to be transferable, and IncI2 and IncX4 are dominant *mcr-1*-carrying plasmid types. In previous studies, IncI2 and IncX4 plasmids harboring *mcr-1* were detected in different species of *Enterobacteriaceae*, owing to the high transfer rate (10^–1^−10^–3^) of *mcr-1* plasmid (Xiaomin et al., 2020). In this study, we also confirmed the p1678-5 plasmid could be transferred from *K. pneumoniae* to *E. coli* in high *in vitro* transfer rate.

According to the related genetic analysis and *in vitro* high conjugation rate, the threat of each single resistant plasmids was verified clearly in *K. pneumoniae* 1678. Once clinical isolate, especially for CRE, uptake one of these plasmids, the infection treatment would be tougher. Previous studies had reported the co-transfer of resistant element, but it is usually associated with one conjugative plasmid (Costa et al., 2021; Gu et al., 2021; Magi et al., 2021). Previous studies found the *mcr-1* plasmid also could co-transfer with *bla*_NDM_ plasmid to one recipient, but these plasmids did not transfer from the same donor like the observation in our study (Liang et al., 2021). Notably, in this study we found the *mcr-1* plasmid (p1678-5) could be co-transferred with *fosA3* plasmid (p1678-3) or *bla*_NDM–5_ plasmid (p1678-4) in 10^–5^ conjugation frequency. Moreover, we also observed these three plasmids could be transferred together, though the transfer rate was not high, the potential risk should be taken seriously. Furthermore, as the overexpression of RND-type efflux transporters (mediating tigecycline resistance) were not rare in *K. pneumoniae* (Bialek-Davenet et al., 2015), once these movable resistant plasmids co-transmit to such isolate, like *K. pneumoniae 1678* in this study, the therapeutic option would be extremely limited. Tigecycline, Fosfomycin, colistin, carbapenems, and ceftazidime–avibactam are considered as the most effective antibiotics to defend XDR isolates, the co-transferring and co-existing of these typical high-risk plasmids would arise a huge peril to clinical treatment since these antibiotics may all be useless.

The dissemination of resistance genes is not only *via* plasmids, but also *via* other mobile structures like transposons and insertion elements. IS*26* plays a key role in the dissemination and mobilization of *fosA3*. This IS*26*-array forms two composite transposons and several IS*26*-based transposition units, and both conformations are capable of transposition and exhibit multiple movement modes. In addition to transposition, gene excision and rearrangement of gene modules *via* homologous recombination between IS*26* scattered in the plasmid and/or genome, also drive the evolutionary process of bacteria (Partridge et al., 2018), which could explain the structure of multiple ORF insertions observed in p1678-3 plasmid. The insertion sequence IS*Aba125* is always upstream of *bla*_NDM_, providing the −35 region of a promoter for the expression of *bla*_NDM_ (Wu et al., 2019). In p1678-4, the Tn*125* was truncated by IS*26* and IS*3000*, forming other composite transposons. The mobilization of *bla*_NDM_ associated with IS*26* or IS*3000* transposons was also common (Wu et al., 2019). These results indicated that although Tn*125* transposon was interrupted, the movability of *bla*_NDM–5_ remained. Previous studies have indicated that IS*Apl1* (always associated with the IncI2 plasmid) is a highly active insertion element and a key component required for the mobilization of the gene-cassette containing the *mcr-1* gene (Xiaomin et al., 2020). However, the IS*Apl1* was absent in the p1678-5 plasmid, and in all the *mcr-1*-carrying IncX4-type plasmids, IS*Apl1* in front of *mcr-1* was lost (Du et al., 2020). The loss of the composite transposon IS*Apl1* might increase the stability of the *mcr* gene in IncX4 plasmids, and promote the widespread dissemination of the *mcr-1* gene.

In this study, we report the coexistence and co-transferring of FosA3-, NDM-5, and MCR-1-encoding plasmids in a *K. pneumoniae* isolate. The co-occurrence of *fosA3*, *bla*_NDM–5_, and *mcr-1*, and the overexpression of RND-type efflux pumps caused 1678 to be highly resistant not only to commonly used antibiotics (e.g., carbapenems, cephalosporins), but also to Fosfomycin, colistin, ceftazidime–avibactam, and tigecycline, which were considered as the last line for defending XDR Gram-negative organisms. Moreover, the high rate of transmission or co-transmission of these plasmids and various mobile elements surrounding resistant genes greatly increased the risk of spread of these resistant phenotypes. The main limitation in this study is we did not apply the conjugation assay between clinical isolates, which means we could not evaluate the dissemination ability of plasmids more accurately, and the *K. pneumoniae* 1678 did not exhibit any hyper-virulent phenotype. However, future studies are still necessary to evaluate the prevalence of such multi-drug resistant isolates.

## Data Availability Statement

The datasets presented in this study can be found in online repositories. The names of the repository/repositories and accession number(s) can be found in the article/Supplementary Material.

## Author Contributions

FY conceptualized and designed the study. YZ performed data analysis and interpretation. YZ, WA, and YC wrote the original draft. YG, XCW, BW, LR, YX, HZ, and XYW contributed to the interpretation of data. All authors contributed to the manuscript drafting.

## Conflict of Interest

The authors declare that the research was conducted in the absence of any commercial or financial relationships that could be construed as a potential conflict of interest.

## Publisher’s Note

All claims expressed in this article are solely those of the authors and do not necessarily represent those of their affiliated organizations, or those of the publisher, the editors and the reviewers. Any product that may be evaluated in this article, or claim that may be made by its manufacturer, is not guaranteed or endorsed by the publisher.

## Footnotes

1
https://github.com/ruanjue/smartdenovo

2
https://cge.cbs.dtu.dk/services/PlasmidFinder/

3
https://cge.cbs.dtu.dk/services/ResFinder/

4
https://tool-mml.sjtu.edu.cn/oriTfinder/oriTfinder.html

5
https://www-is.biotoul.fr/
