# Supplementary material for: The Co-occurrence of NDM-5, MCR-1, and FosA3-Encoding Plasmids Contributed to the Generation of Extensively Drug-Resistant Klebsiella pneumoniae
Source: Front Microbiol. 2022 Jan 3;12:811263. doi: 10.3389/fmicb.2021.811263 (PMC8762306; doi:10.3389/fmicb.2021.811263)
Supplement: Supplementary file 1 [file Data_Sheet_1.DOCX]

**Supplementary materials**

**Table. S1 Oligonucleotides for PCR**

**Fig. S1 The comparison of amino-acid sequence of the Tet(A) protein of *K. pneumoniae* 1678 with reference sequence and other mutated Tet(A) protein.**

**Fig. S2 The tet(A) mRNA expression in original *K. pneumoniae* 1678 and its *tet(A)*** ***E. coli* transformants.**

**Fig. S3 Comparative analysis of pl678-6 plasmid with other reference plasmids.**

**Fig. S4 Comparative analysis of pl678-5 plasmid with pHNSHP45 plasmid.**

**Table.S1 Oligonucleotides for PCR**

| **Name** | **Sequence** |
| --- | --- |
| **For resistant genes confirmation** |  |
| *bla*_NDM-5_-F | GTCTGGCAGCACACTTCCTA |
| *bla*_NDM-5_-R | GCGGGCCGTATGAGTGATT |
| *fosA3*-F | TCAGCGATCTGGCGTCAAG |
| *fosA3*-R | ATGCAGCTCCAGCTTATGGC |
| *mcr-1*-F | AAACCTATCCCATCGCGGAC |
| *mcr-1*-R | AGCTGAACATACACGGCACA |
| *tetA*-F | CGCATAGATCGCCGTGAAGA |
| *tetA*-R | GCTTCATGAGCGCCTGTTTC |
| **For qPCR** |  |
| rrsE-F (Housekeeping gene) | CTACAATGGCATATACAA |
| rrsE-R (Housekeeping gene) | TTCTGATCTACGATTACT |
| qramA-F | ATTTCCGCTCAGGTGATT |
| qramA-R | GTTGCAGATGCCATTTCG |
| qAcrB-F | TGTGCCCTGTCGGTAATGAA |
| qAcrB-R | AACGCGCCATCAGAACAAAC |
| qAcrA-F | GGCAAACATGGATCAACTG |
| qAcrA-R | GGCGGTATCGTAGTCTTG |
| qTolc-F | CTACGCTGTATAACGCTAA |
| qTolc-R | CTAACGCCGACTTAATGT |
| qrarA-F | GACCATCCTGTTTGTTGAC |
| qrarA-R | GTGCCGTCTTCAATATGC |
| qOqxB-F | ATCAGGCGCAGGTTCAGGT |
| qOqxB-R | CGCCAGCTCATCCTTCACTT |
| qOqxA-F | CGCAGCTTAACCTCGACTTCA |
| qOqxA-R | ACACCGTCTTCTGCGAGACC |

**
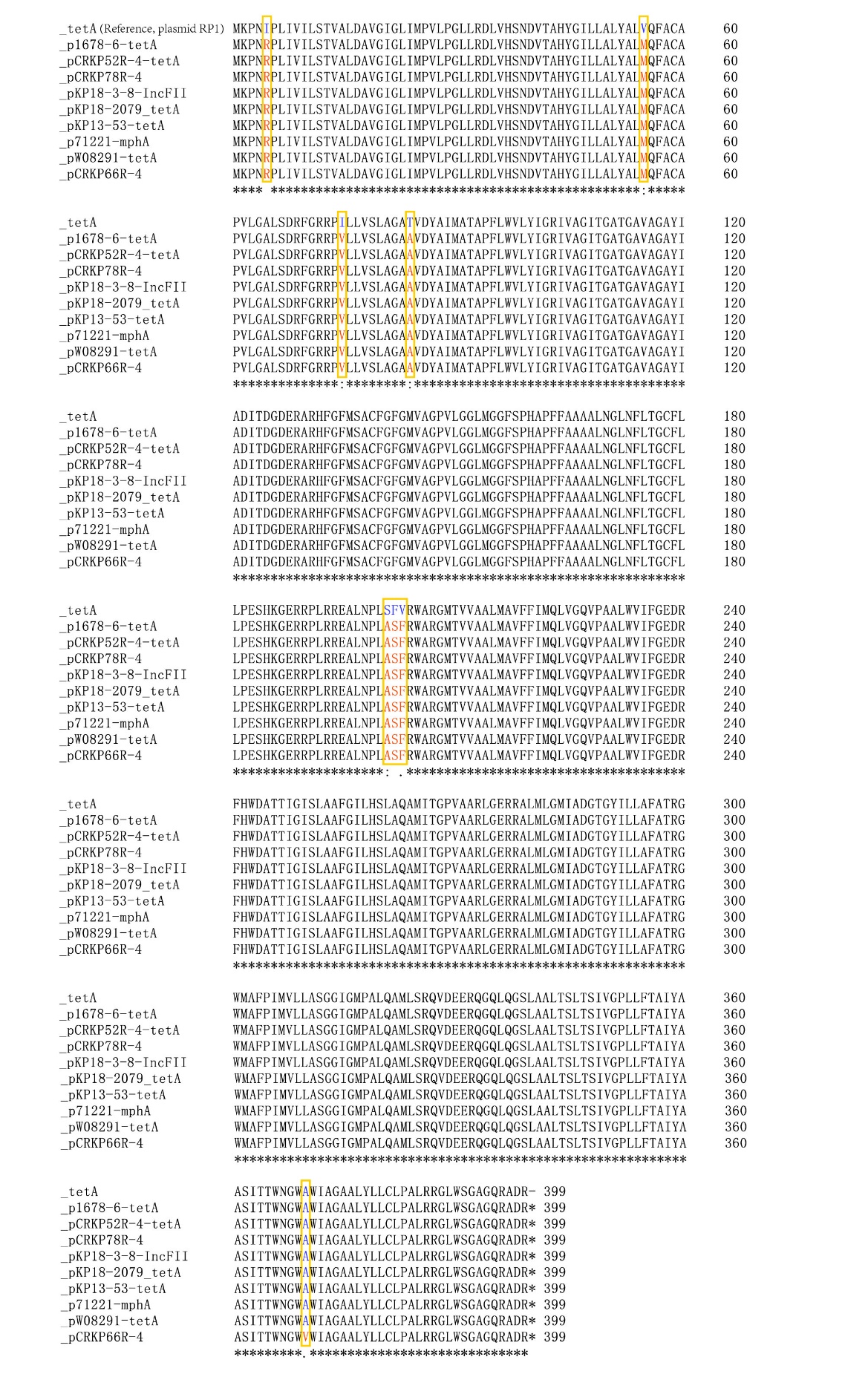
**

**Fig.S1 The comparison of amino-acid sequence of the Tet(A) protein of *K. pneumoniae* 1678 with reference sequence and other mutated Tet(A) protein.** The position of mutated amino-acid was marked with yellow box and red font. The reference Tet(A) protein was extracted from plasmid RP1 (Accession no. X00006). The other mutated Tet(A) proteins were reported previously(Xu et al., 2021).


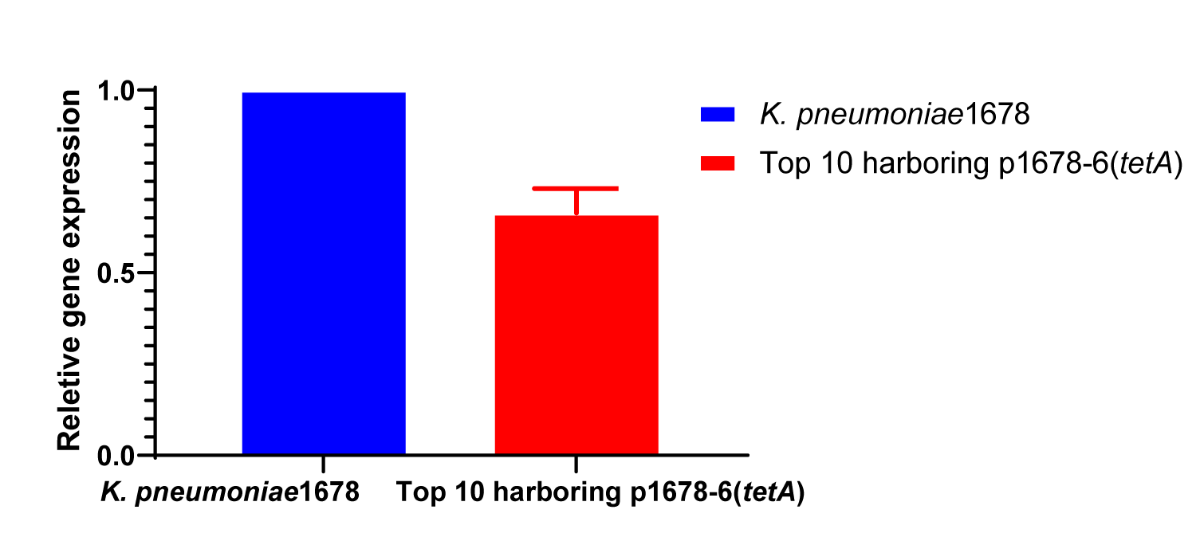


**Fig. S2 The *tet(A)* mRNA expression in original *K. pneumoniae* 1678 and its *tet(A) E. coli* transformants.**


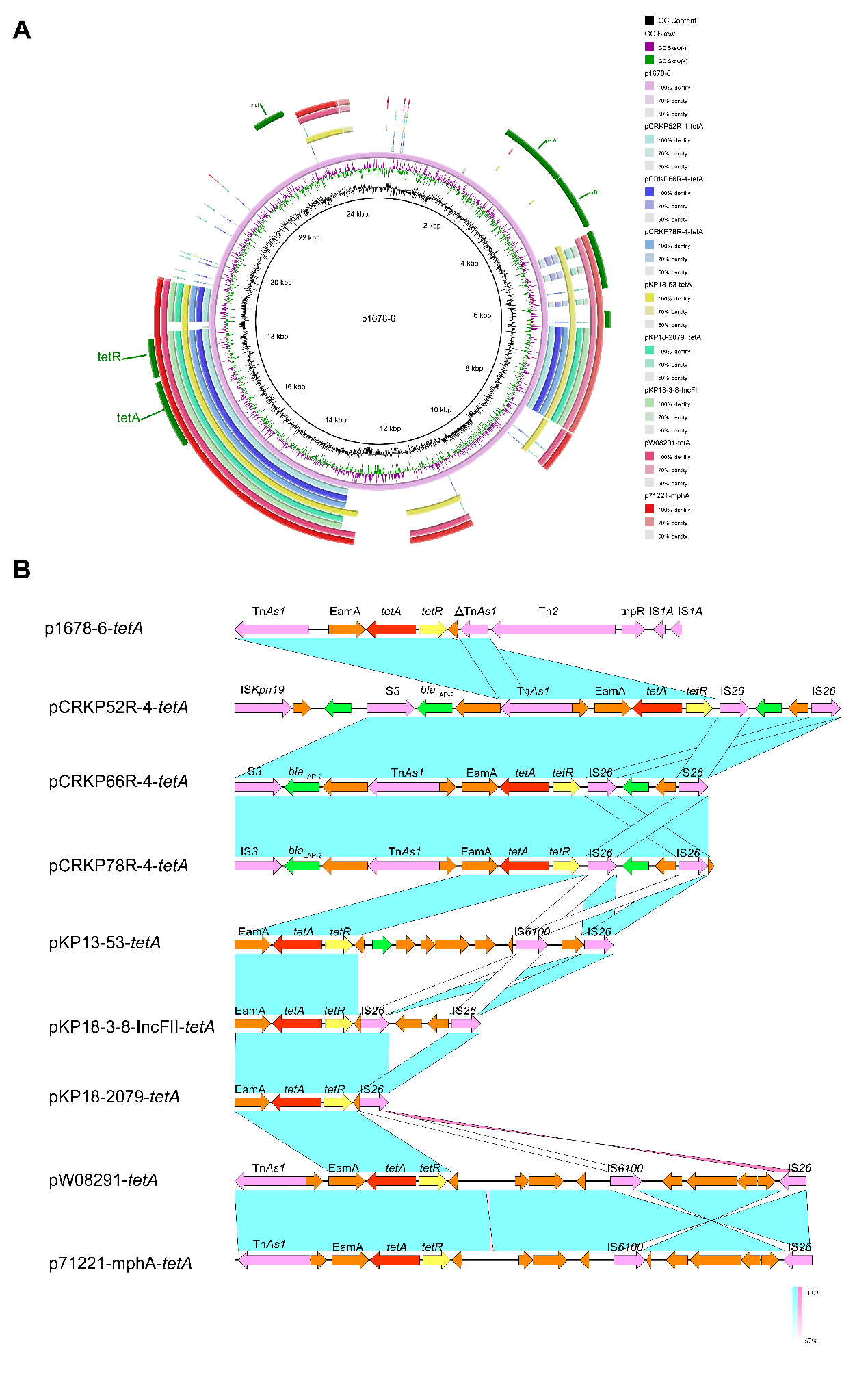


**Fig.S3 Comparative analysis of pl687-6 plasmids with other reference plasmids.** (A) p1678-6 (CP080450) was used as the reference plasmid to perform genome alignment with pCRKP52R-4-tetA (CP066252), pCRKP66R-4-tetA (CP063836), pCRKP78R-4-tetA (CP066257), pKP18-3-8-IncFII (MT035876), pKP18-2079_tetA (MT090960), pKP13-53-tet(A) (MN268580), p71221-tetA (MN310374), and pW08291-tetA (MN310376)(Xu et al., 2021). (B) Linear comparison of the *tetA* region of those *tetA* positive plasmids in (A).


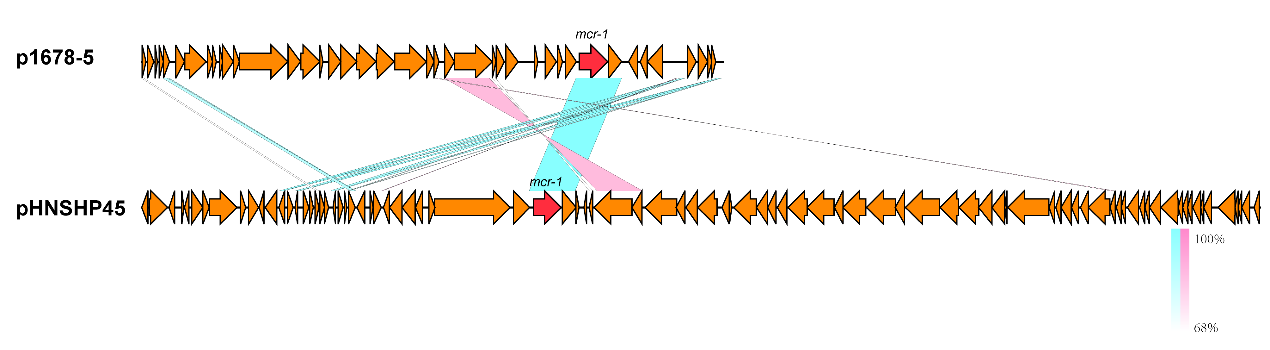


**Fig.S4 Comparative analysis of pl678-5 plasmid with pHNSHP45 plasmid.**

p1678-5 (CP080449) was used as the reference plasmid to perform genome alignment with pHNSHP45(NZ_KP347127.1)(Liu et al., 2016).

**References**

1. Xu, J., Zhu, Z., Chen, Y., Wang, W., and He, F. (2021). The Plasmid-Borne tet(A) Gene Is an Important Factor Causing Tigecycline Resistance in ST11 Carbapenem-Resistant Klebsiella pneumoniae Under Selective Pressure. Front. Microbiol. 12. doi: 10.3389/fmicb.2021.644949

2. Liu, Y. Y., Wang, Y., Walsh, T. R., Yi, L. X., Zhang, R., Spencer, J., Doi, Y., Tian, G., Dong, B., Huang, X., Yu, L. F., Gu, D., Ren, H., Chen, X., Lv, L., He, D., Zhou, H., Liang, Z., Liu, J. H., and Shen, J. (2016). Emergence of plasmid-mediated colistin resistance mechanism MCR-1 in animals and human beings in China: a microbiological and molecular biological study. Lancet Infect. Dis. 16(2), 161-8. doi: 10.1016/S1473-3099(15)00424-7
